# How Well Can Saliency Models Predict Fixation Selection in Scenes Beyond Central Bias? A New Approach to Model Evaluation Using Generalized Linear Mixed Models

**DOI:** 10.3389/fnhum.2017.00491

**Published:** 2017-10-31

**Authors:** Antje Nuthmann, Wolfgang Einhäuser, Immo Schütz

**Affiliations:** ^1^Department of Psychology, School of Philosophy, Psychology and Language Sciences, University of Edinburgh, Edinburgh, United Kingdom; ^2^Perception and Cognition Group, Institute of Psychology, University of Kiel, Kiel, Germany; ^3^Physics of Cognition Group, Institute of Physics, Chemnitz University of Technology, Chemnitz, Germany

**Keywords:** naturalistic scenes, eye movements, saliency models, evaluation metrics, fixation probability, GLMM

## Abstract

Since the turn of the millennium, a large number of computational models of visual salience have been put forward. How best to evaluate a given model's ability to predict where human observers fixate in images of real-world scenes remains an open research question. Assessing the role of spatial biases is a challenging issue; this is particularly true when we consider the tendency for high-salience items to appear in the image center, combined with a tendency to look straight ahead (“central bias”). This problem is further exacerbated in the context of model comparisons, because some—but not all—models implicitly or explicitly incorporate a center preference to improve performance. To address this and other issues, we propose to combine *a-priori* parcellation of scenes with generalized linear mixed models (GLMM), building upon previous work. With this method, we can explicitly model the central bias of fixation by including a central-bias predictor in the GLMM. A second predictor captures how well the saliency model predicts human fixations, above and beyond the central bias. By-subject and by-item random effects account for individual differences and differences across scene items, respectively. Moreover, we can directly assess whether a given saliency model performs significantly better than others. In this article, we describe the data processing steps required by our analysis approach. In addition, we demonstrate the GLMM analyses by evaluating the performance of different saliency models on a new eye-tracking corpus. To facilitate the application of our method, we make the open-source Python toolbox “GridFix” available.

## Introduction

Computational models of visual attention are used to derive predictions about the spatial distribution of eye fixations in a scene, which can then be compared to observed human fixations. An important issue concerns the methods that are used to determine how well a given model captures human behavior (Wilming et al., 2011; Borji et al., 2013a; Bylinskii et al., 2015). The goal of the present article is to present a new method that addresses a number of key issues that have made model evaluation and comparison challenging in the past.

Models of visual attention (Frintrop et al., 2010, for review) can be grouped as models that emphasize either bottom-up (or stimulus-driven) or top-down (or context-driven, or goal-driven) guidance of attention (cf. Itti and Borji, 2014). In this context, bottom-up guidance is essentially used synonymously to “salience.” Intuitively described, salience defines how much an element of the scene, be it an object or a region, stands out from its neighboring parts (Borji and Itti, 2013). Bottom-up models typically use low-level features in the process of building a saliency map, as initially proposed by Koch and Ullman (1985). The first implementation of a saliency map was provided by Itti et al. (1998). Since then, the notion of bottom-up attention and visual salience has given rise to many new computational models (Rothenstein and Tsotsos, 2008; Borji and Itti, 2013; Kimura et al., 2013, for reviews). The architecture of many saliency map models is influenced by the neurobiology and psychophysics of human and primate vision. A current trend is the emergence of saliency models with deep learning architectures, which outperform traditional models (Bylinskii et al., 2016b). Review chapters by Riche and Mancas provide brief systematic descriptions of bottom-up saliency models for static (Riche and Mancas, 2016a) and dynamic (Riche and Mancas, 2016b) images.

An attractive feature of saliency map models is that, once implemented, they are “image-computable.” That is, they can be applied to any image and produce output that can be tested against experimental data. Under the assumption that visual salience guides where observers look in an image, eye movement data are frequently used to validate predictions of bottom-up saliency models. Thus, one would compare the fixation data recorded from observers viewing the same stimuli as given to the model to the saliency map produced by the model.

Given the ever-increasing number of saliency models that are being developed, the challenge is to determine which model(s) provide(s) the best approximation to human observers' eye fixations. In fact, Itti and Borji (2014) conclude in their review that “carrying out standardized evaluations is important to ensure that the field keeps moving forward” (p. 1147). Various metrics have been used for model evaluation and comparison. Borrowed from signal detection theory (Green and Swets, 1966), the Area Under the Receiver Operating Characteristics (ROC) Curve, referred to as AUC, is the most widely used metric for evaluating saliency maps (e.g., Borji et al., 2013a). There are different implementations available dealing with some limitations of the classical approach (Riche, 2016b, for review). Other metrics include Spearman's Correlation Coefficient (Toet, 2011), Pearson's Correlation Coefficient (Ouerhani et al., 2004), Normalized Scanpath Salience (Peters et al., 2005), Kullback-Leibler Divergence (Kullback and Leibler, 1951; Rajashekar et al., 2004), Earth-Mover's Distance (Rubner et al., 1998; Pele and Werman, 2008; Judd et al., 2012b), and a measure of information gain (Kümmerer et al., 2015).

A detailed discussion of the metrics used in saliency model evaluation is provided elsewhere (Wilming et al., 2011; Emami and Hoberock, 2013; Le Meur and Baccino, 2013; Riche et al., 2013; Sharma, 2015; Bylinskii et al., 2016a; Riche, 2016b). Bylinskii et al. (2016a) review a number of recent papers that have compared different saliency models across different metrics and datasets (Toet, 2011; Zhao and Koch, 2011; Borji et al., 2013a,b; Emami and Hoberock, 2013; Le Meur and Baccino, 2013; Riche et al., 2013; Li et al., 2015; see also Sharma, 2015; Riche, 2016c).

Assessing the performance of visual salience algorithms is not without challenges (Bruce et al., 2015; Rahman and Bruce, 2015). Borji et al. (2013a) contemplate: “Perhaps the biggest challenge in model comparison is the issue of center-bias.” (p. 59). The central bias of fixation describes the well-established finding that observers fixate more often toward the center of the image than its edges (Mannan et al., 1996; Parkhurst and Niebur, 2003; Tatler et al., 2005; Clarke and Tatler, 2014; Nuthmann and Einhäuser, 2015). Interestingly, there is also a feature bias such that most photographs of scenes have a bias toward higher salience in the center than around the edges of the images (“photographer bias”). Tatler (2007) demonstrated that the central fixation bias in scene viewing is not explained by such centrally located features; this implies that the coincidence of central fixation bias and photographer bias remains a possible confound for the evaluation of saliency models. Regarding model evaluation, the best solution to the issue of center bias is to design suitable evaluation metrics (Borji et al., 2013a), an approach we adopt here.

A critical aspect of model evaluation and comparison is that scores are typically based on average performance over a dataset of images (Itti and Borji, 2014, for discussion). The dataset (Riche, 2016a, for a survey) is often hand-picked, and it may contain significant biases (Torralba and Efros, 2011). Average measures can be dominated by trivial cases. Not only that, departure from average performance may provide important diagnostic information for model development (Kümmerer et al., 2015). To address these issues, we propose a method that can capture whether scene items vary in the extent to which image salience affects fixation selection.

As a new model evaluation method, we propose to combine *a-priori* parcellation of scenes with generalized linear mixed models (GLMM). This approach builds upon our previous work in which we combined a scene-patch analysis with GLMM (Nuthmann and Einhäuser, 2015). The scene-patch analysis allowed for describing the relationship between (continuous) image feature values and fixation probability. GLMM were used to estimate the unique contribution of various image features to fixation selection: luminance and luminance contrast (low-level features); edge density (a mid-level feature); visual clutter and image segmentation to approximate local object density in the scene (higher-level features). The GLMM results revealed that edge density, clutter, and the number of homogenous segments in a patch can independently predict whether image patches are fixated or not. Importantly, neither luminance nor contrast had an independent effect above and beyond what could be accounted for by the other image features.

Our previous work in Nuthmann and Einhäuser (2015) can be described as addressing the question of what features should be part of the saliency map. Here, we adopt this approach to evaluate how well a given saliency map model predicts where human observers fixate in naturalistic images, above and beyond what can be accounted for by the central bias of fixation. Moreover, we can assess directly whether a given saliency model performs significantly better than others. In the GLMMs, by-subject and by-item random effects account for individual differences and differences across scene items.

In the remainder of the article, we first describe the data processing steps required by our analysis approach. Next, we demonstrate the GLMM analyses by evaluating the performance of different saliency models on a new eye-tracking corpus. To facilitate the application of our method, we provide the open-source Python toolbox GridFix which performs the data processing steps that are needed for the GLMM analyses.

## Materials and methods

### Participants, apparatus, and materials

Analyses were based on data from a new corpus of eye movements during scene viewing and sentence reading. We analyzed the scene-viewing data from 42 young adults (8 men and 34 women) between the ages of 18 and 29 years (mean age = 22.1 years). The young participants were students at the University of Edinburgh. Another 34 older adults from the community contributed to the eye-movement corpus; the 17 men and 17 women averaged 72.1 years of age (range = 66 years to 83 years). The data from the older participants were not included in the present analyses. All participants had normal or corrected-to-normal vision. They received monetary compensation for their participation. The study was carried out in accordance with the recommendations of the Psychology Research Ethics Committee of the University of Edinburgh with written informed consent from all subjects. All subjects gave written informed consent in accordance with the Declaration of Helsinki. The protocol was approved by the Psychology Research Ethics Committee of the University of Edinburgh.

Each participant viewed 150 color photographs of real-world scenes, which were presented in random order to them. Most of these images (136) depicted indoor scenes, which were rich in objects and relatively cluttered. The scene images were chosen such that they were also suitable for a related study in which a new set of young and older adults searched for a pre-defined object in the scene. Scenes were presented on a 21-inch CRT monitor with a screen resolution of 800 × 600 pixels and subtended 25.78° horizontally × 19.34° vertically at a viewing distance of 90 cm. Eye movements were recorded using an SR Research EyeLink 1000 Desktop mount system. It was equipped with the 2,000 Hz camera upgrade, allowing for binocular recordings at a sampling rate of 1,000 Hz for each eye. Data from the right eye were analyzed. The experiment was implemented with the SR Research Experiment Builder software.

### Design and procedure

Each trial started with a centrally located pre-trial fixation cross, which acted as a fixation check. Afterwards, the scene was presented for 6 s. Participants were instructed to commit the scene to memory. To probe participants' scene encoding, test questions were pseudo-randomly distributed throughout the experiment and occurred on 30 of the trials. These questions examined memory for specific objects in the scenes (e.g., “Was there a book?”).

### Data analysis

Gaze raw data were converted into a fixation sequence matrix using SR Research Data Viewer. Saliency maps were computed in MATLAB 2014a (The MathWorks, Natick, MA). Data processing for this article was programmed in MATLAB. The code was then re-implemented and generalized in the new open-source toolbox GridFix^1^, which was programmed in Python (version 3.4; http://python.org). Statistical analyses were performed using the R system for statistical computing (version 3.2.2; R Development Core Team, 2015). For GLMMs we used the *glmer* program of the *lme4* package (version 1.1-10, Bates et al., 2015b) supplied in *R*. By default, *glmer* uses a combination of Nelder-Mead and bobyqa optimizers.

#### Overview of analysis steps

**Figure 1:**
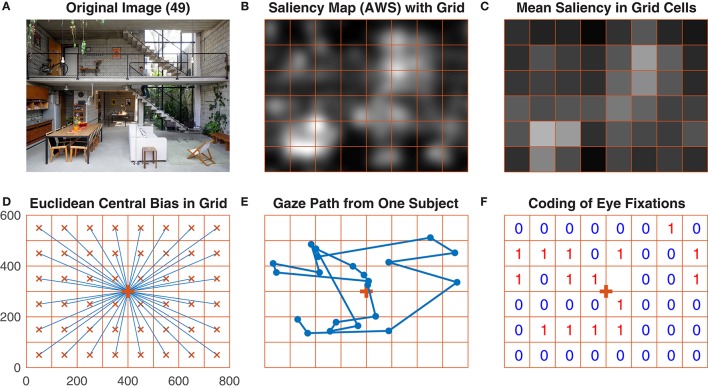
Main steps of data processing performed by the GridFix toolbox. **(A)** One of the images used in the study. **(B)** A saliency map (Adaptive Whitening Saliency) for this image, with the analysis grid overlaid. Note that brighter areas indicate higher visual salience. **(C)** Mean saliency values for the grid cells, color-coded. **(D)** Image grid with vectors (in blue) connecting the center of the grid cell with the center of the image. **(E)** Gaze path from one subject over the image in **(A). (F)** Coding of this subject's eye fixations in the image grid.

We propose a new method for the quantitative evaluation of saliency models, which requires four analysis steps (see Figure 1). First, for each image one or more saliency maps are constructed via image processing. To this end, researchers use software and code made available by the authors of published saliency models. In the GridFix toolbox, collections of images (scenes and/or saliency maps) are represented as an *ImageSet* object, which assigns a unique *image ID* to every image. Through this ID, each scene image is associated with the corresponding saliency maps as well as the fixation data. Moreover, it allows the user to easily adapt the same analysis to different sets of images/maps, simply by exchanging objects. For the present analyses, the saliency map of each image was normalized to the same range, arbitrarily chosen as [0,1]; the GridFix toolbox provides both original as well as normalized values.

In the second step, each photograph is parcellated into local image regions. For the present example analyses, we use an 8 × 6 grid yielding 48 quadratic scene patches, with each grid cell spanning 3.2° × 3.2° (100 × 100 pixels). For each patch, a local saliency value is then extracted from a given saliency map. Local saliency is defined as the mean over the saliency map's values within each grid cell. Users of the GridFix toolbox need to specify both the image size and the grid cell size. GridFix then generates a regular grid as a set of binary masks, which are stored as a *RegionSet* object and used to select the corresponding pixels from a given image or saliency map. This allows for easy comparison of results for different grid sizes for example, simply by changing the parcellation objects and by recomputing the predictor matrix.

Third, the empirical eye-fixation data are mapped onto the scene analysis grid. This requires constructing an observation matrix based on the experimental design and the fixation data. For the present data, the complete observation matrix would comprise 302,400 rows (150 images × 42 subjects × 48 grid cells). However, there were eight missing trials. Accordingly, the GridFix toolbox creates the observation matrix based on trials for which fixation data exist (302,016 rows). For each observer, image, and image patch, GridFix can calculate two categorical response variables on demand. A binary variable is coding whether a given image patch was fixated (1) or not (0) throughout the trial. In addition, a count variable records the total number of fixations that fell on a given scene region throughout the trial (incl. immediate refixations and later revisits).

Fourth, we use GLMM to assess the impact of visual salience on selecting image patches for fixation. To facilitate the statistical analyses, GridFix outputs the GLMM predictors as a comma-separated file that can be loaded into R. GridFix also generates basic R source code to load the toolbox output and to analyze the data using GLMM. This code is provided to facilitate initial explorations; in most cases, it will need to be adjusted to the individual data and design as appropriate.

#### Computation of image salience

For each image, three different saliency maps were computed. First, we used Itti et al.'s (1998) implementation of Koch and Ullman's (1985) computational architecture extending the Feature Integration Theory (Treisman and Gelade, 1980). Specifically, we used the MATLAB code that is most faithful to the original model and its parameters as provided as the “simpsal” model in the graph-based visual saliency (GBVS) paper (Harel et al., 2007). We refer to this model as IKN98.

Second, we used the Adaptive Whitening Saliency (AWS) model, which is based on the variability in local energy as a measure of salience (Garcia-Diaz et al., 2012a,b). The AWS model was chosen because it was ranked first in its ability to predict where observers look in static and dynamic natural scenes in a recent comparative study (Borji et al., 2013a). We used the MATLAB implementation provided by the authors at http://persoal.citius.usc.es/xose.vidal/research/aws/AWSmodel.html, using a scaling factor of 1.0 to the unmodified version of each image in its pixel intensity representation. Except for the scaling factor, which has a default value of 0.5 to reduce computation time for large images, default parameters as set in the authors' implementation were used.

Third, we used the graph-based visual saliency (GBVS) model (Harel et al., 2007), with source code downloaded from http://www.vision.caltech.edu/~harel/share/gbvs.php. The model was introduced as an alternative to the then standard IKN98 model. The GBVS model consists of two components: a measure of local dissimilarity and a directed graph between local nodes. In a first step, local activations are computed based on local dissimilarity, in a second “normalization” step activity is concentrated on a few nodes, effectively sparsifying the resulting salience representation. Equating the nodes with image locations, GBVS predicts fixation patterns on natural scenes. Unlike IKN98 and AWS, GBVS has an intrinsic central bias, as the inner nodes have more connections to other nodes than the nodes toward the image boundaries. Thus, the salient regions at the center of the image receive more weight than the peripheral regions. The built-in central bias makes GBVS an interesting test case for our model evaluation method.

#### Central bias

A major advantage of our evaluation method for saliency models is that we can explicitly model the central bias of fixation by including a central-bias predictor in the GLMM. Motivated by previous research, GridFix provides different alternatives for both Euclidean as well as Gaussian distance-to-center variables (Clarke and Tatler, 2014, for review). Moreover, we provide the taxicab distance as a natural alternative when using a grid.

**Figure 2:**
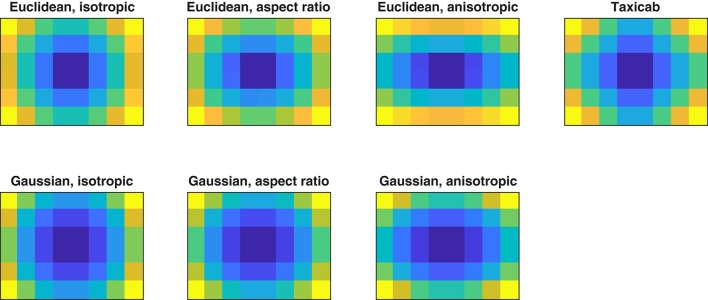
Visualization of the central-bias variables calculated by the GridFix toolbox. Color coding indicates normalized distance of corresponding cell centers to image center (brighter is more peripheral; dark blue: smallest distance, normalized to 0; yellow: largest distance, normalized to 1).

In the GridFix toolbox, central bias is treated in the same way as local image features: a local feature value for each grid cell is calculated, indicating the distance of each cell's center to the center point of the image. These distance values can be added as a predictor to the GLMM. Upon creation of a *CentralBiasFeature* object, the user can specify which distance measure to use (i.e., “euclidean,” “gaussian,” or “taxicab”) along with further parameters (e.g., for anisotropy; see below). Table 1 summarizes the taxonomy of the central-bias variables. Moreover, Table 1 includes the formulas for calculating the different distance-to-center variables, and Figure 2 provides a visualization.

**Table 1:** Taxonomy of central-bias variables calculated by the GridFix toolbox.

	**Isotropic**	**Aspect ratio**	**Anisotropic**
Euclidean	$C_{g,euclidean}=\sqrt{{\left(x_{g}-x_{c}\right)}^{2}+{\left(y_{g}-y_{c}\right)}^{2}/\nu }$		
Parameters	ν = 1	ν = *h*/*w* = 0.75	ν = 0.45
Rank	5	2	1
Gaussian	$C_{g,gaussian}=-\exp\left[-\left(\frac{{\left(x_{g}-x_{c}\right)}^{2}}{2\sigma_{x}^{2}}\right)-\left(\frac{{\left(y_{g}-y_{c}\right)}^{2}}{2\sigma_{x}^{2}\nu }\right)\right]$		
Parameters	$\sigma_{x}^{2}=0.23,\;\nu =1$	$\sigma_{x}^{2}=0.23,\;\nu =0.75$	$\sigma_{x}^{2}=0.23,\;\nu =0.45$
Rank	7	6	4
Taxicab	*C*_*g,taxicab*_ = |*x*_*g*_ − *x*_*c*_| + |*y*_*g*_ − *y*_*c*_|		
Rank	3		

*Formulas indicate how the distance between the center of each grid cell (x_g_, y_g_) and image center (x_c_, y_c_) is calculated for each variable. h/w, height/width of image. For additional parameters ($\sigma_{x}^{2}$, horizontal variance for Gaussian variables; ν, anisotropy parameter), we used the values suggested by Clarke and Tatler (2014) as a default. Rank numbers represent the ordering of central-bias predictors according to GLMM analyses presented in Figure 4*.

For each of the Euclidean distance-to-center variables, GridFix determines the distance between the center of the respective grid cell and the center of the image (blue vectors in Figure 1D, see also Figure 2 in Nuthmann and Einhäuser, 2015). Similarly, the “taxicab” predictor is generated using the distance between each cell center and the center of the image along the horizontal and vertical image axes. This distance is called taxicab or Manhattan distance because it is the distance a taxi would drive in a city like Manhattan where the buildings are laid out in square blocks and the straight streets intersect at right angles (Craw, 2010). Alternatively, the user can choose a Gaussian central-bias variable. For each grid cell the distance to center is read off from a two-dimensional Gaussian distribution which is centered over the midpoint of the grid.

Using the definitions above, the Euclidean and Gaussian central-bias variables correspond to isotropic measures, assuming equal spread of fixation positions in the horizontal and vertical dimensions. For example, Zhao and Koch (2011) fitted an isotropic Gaussian to their fixation data to account for central bias, i.e., they fitted a covariance matrix with equal horizontal and vertical variance. GridFix offers two additional alternatives. First, the Euclidean or Gaussian distance-to-center measure can be scaled to the aspect ratio of the image. This option is motivated by previous research in which a Gaussian scaled to the aspect ratio of the images was used (Judd et al., 2012b). Second, GridFix provides an optional anisotropy parameter *v*, which acknowledges that fixation positions in scene viewing typically show a greater spread of fixations horizontally than vertically (Clarke and Tatler, 2014).

To calculate Gaussian central-bias input variables GridFix needs parameter values that describe the variance of the two-dimensional Gaussian distribution. The default values correspond to the recommendations by Clarke and Tatler (2014). Specifically, the parameter for the variance of the Gaussian defaults to σ^2^ = 0.23 (in units of half the image width). For the anisotropic Gaussian and Euclidean measures, the scaling parameter for the vertical dimension defaults to *v* = 0.45^2^. Thus, for the anisotropic Gaussian central-bias input variable the vertical variance *v*σ^2^ is set to 0.10 (0.45 ^*^ 0.23). Of course, users can also derive these parameter values from their own data—following the procedure in Clarke and Tatler (2014)—and submit them as arguments to GridFix.

We note that aspect-ratio variables and anisotropic variables are identical if *v* equals the inverse of the aspect ratio (e.g., *v* = 0.75 for aspect ratio 4:3). To ease comparison with a given Euclidean distance-to-center predictor, in the GLMMs any Gaussian central-bias predictor should be entered with a negative sign, such that increasing values correspond to more peripheral locations. GridFix does this automatically such that distance values do not need to be inverted.

#### Generalized linear mixed models

Generalized linear mixed models (Barr, 2008; Jaeger, 2008; Bolker et al., 2009; Moscatelli et al., 2012; Agresti, 2013; Demidenko, 2013) are used to determine the impact of image salience on fixation probability in scenes. An advantage of GLMM is that they avoid information loss due to prior averaging over items or subjects; hence we can model the data at the level of individual observations. The probabilities and/or counts are modeled through a link function. For binary data (1 fixated, 0 not fixated), there are three common choices for link functions: logit, probit, and complementary log-log (Agresti, 2013; Demidenko, 2013). For the present analyses, we use the logit transformation of the probability, which is the default for *glmer*. Thus, in a binomial GLMM parameter estimates are obtained on the log-odds or logit scale, which is symmetric around zero, corresponding to a probability of 0.5, and ranges from negative to positive infinity. Consequently, negative log-odds correspond to probabilities *p* < 0.5. On the other hand, fixation count data are multinomial response variables, which are modeled using a poisson GLMM with a log link function (Agresti, 2013). For the GLMMs we report regression coefficients (*b*s), standard errors (SEs), and *z*-values (*z* = *b*/SE).

The input variables (i.e., the variables that are measured) and predictors (i.e., the terms that are entered into the GLMM) of interest in this study are saliency and central bias, both of which were measured on a continuous scale. For the GLMM analyses, both input variables were centered by subtracting the sample mean from all variable values and scaled by dividing the variables by their sample standard deviations. As a result, the input variable had a mean of 0 and a standard deviation of 1. This standardization (*z*-transformation) converts the original units to units of standard deviations. In the case of approximately normal distributed input variables, about 95% of the values are within ±2 units. Standardization of input variables results in the estimation of standardized slopes, which are comparable in magnitude within models as well as between models (Schielzeth, 2010). In GridFix, unstandardized variables are exported to R, but the generated R source code by default includes code to standardize all input variables before inclusion in the GLMM.

Mixed models are statistical models that incorporate both fixed effects and random effects (Bates, 2010). The fixed effects of interest in the present context are local saliency and central bias, along with the intercept. Wald *z*-tests for GLMMs test the null hypothesis of no effect by scaling parameter estimates by their estimated standard errors and comparing the resulting *z*-statistic to zero (Agresti, 2013). The intercept represents the overall fixation probability, describing the proportion of scene patches observers selected for fixation. A significant positive slope for saliency is indicative of a reliable impact of image salience on fixation probability. Similarly, a significant negative slope for central bias substantiates that fixation probability decreases with increasing distance from image center.

Random effects represent subjects' or items' deviations from the fixed-effect parameters (Bates, 2010). The intercept has two random components, one varying from subject to subject and one varying from scene item to scene item. The random intercepts allow for the fact that some observers sample more scene patches on average than others, and their fixation coverage is higher (on average) for some scenes than for others. In principle, the variance-covariance matrix of the random effects not only includes random intercepts but also random slopes as well as correlations between intercepts and slopes. Random slopes account for variance between subjects and between items for fixed effects in the GLMM. Including by-subject random effects is also a way of accounting for individual differences (Kliegl et al., 2011). For example, by including a by-subject random slope for local saliency we can assess the degree to which subjects vary in their response to image salience. Similarly, the by-item random slope for local saliency captures whether scene items vary in the extent to which image salience affects fixation selection. The by-item random slope for central bias describes whether scene items differ in eliciting more eye fixations at the center of the image compared to the periphery. The maximal random-effects structure also includes correlations between variance components. To give an example, we may expect a correlation between the random intercept and the random slope for central bias—both for scene items and observers—such that the more patches were fixated, the smaller the central fixation bias (Nuthmann and Einhäuser, 2015).

The selection of an appropriate random-effects structure requires some care. The random intercept model has the simplest structure in that it includes random intercepts for subjects and items only. When random variances are present in the underlying populations, not including corresponding random slopes in the statistical model inflates Type I error rates (Schielzeth and Forstmeier, 2009). Thus, the researcher may conclude that a supposed effect exists, when in fact it does not. Therefore, some authors recommend that all models should be “maximal,” with all possible random slopes and correlation parameters included (Barr et al., 2013). However, if the variances of some random effects are very small, fitting a maximal model can lead to a significant loss of statistical power (Matuschek et al., 2017). Thus, the assumed protection against Type I errors can come at the cost of a considerable increase in Type II error rate. Moreover, the number of model parameters associated with random factors grows quadratically with the number of variance components. Thus, for more complex designs the maximal random-effects structure may be too complex for the information contained in the data (Bates et al., 2015a). If the maximal model is overparameterized or degenerate relative to the information in the data, it typically fails to converge.

How can model complexity be reduced without taking the risk of inflating the Type I error? One recommendation is to replace the maximal model with a zero-correlation parameter (zcp) model in which the random slopes are retained but the correlation parameters are set to zero (Barr et al., 2013). Thus, in the zcp model random slopes and intercepts are assumed to be independent (Barr et al., 2013; Bates et al., 2015b). A perhaps more elaborate alternative is to build a “parsimonious” model, which contains only variance components and correlation parameters that are supported by the data (Bates et al., 2015a). Parsimonious models appear to improve the balance between Type I error and statistical power (Matuschek et al., 2017).

A parsimonious model can be determined by using a standard model selection criterion. Common model selection criteria are the likelihood ratio test (LRT), the Akaike Information Criterion (AIC, Akaike, 1973), and the Bayesian Information Criterion (BIC, Schwarz, 1978). The LRT compares two different models to determine if one is a better fit to the data than the other. In the present context, LRTs are used to decide if a particular random effect should be included in the model by evaluating, for example, whether dropping that effect from the maximal model leads to a significantly worse fit of the model. The log-likelihood increases with goodness of fit. The AIC (Burnham et al., 2011, for review) corrects the log-likelihood statistic for the number of estimated parameters. The BIC additionally corrects for the number of observations. The AIC and BIC both decrease with goodness of fit.

Random effects and their correlations can be tested using backward or forward model selection (Barr et al., 2013). Backward selection starts with the maximal or most complex model, whereas forward selection typically starts with a random-intercepts-only model. Whether random effects are warranted is essentially an empirical question (Judd et al., 2012a); there is no one-size-fits-all solution, and this is why the GridFix toolbox only generates basic R code to facilitate initial explorations. As a rule of thumb, model complexity can be reduced by removing parameters that are zero (or very close to zero) and by removing nonsensical estimates of correlation parameters (i.e., values of −1 or +1). In principle, any of the special cases discussed above (random intercept model, zcp model, maximal model) could be identified as a parsimonious model.

We conclude this section by previewing our model selection strategy for the results reported below. For the one-predictor GLMMs (step 1) we report maximal GLMMs. For the Central-Bias—Saliency GLMMs (step 2), each testing one of the saliency maps, we report maximal models as well (with one exception, see Footnote 4). Exploring these two-predictor GLMMs further, the results section concludes with a comprehensive set of control analyses, part of which were designed to compare the maximal models with corresponding zcp models and random intercept models. When using one of the Central-Bias—Saliency GLMMs to discuss individual differences and item effects (step 3), we explicitly compare the maximal model to both the zcp model and the random intercept model. For the most complex Central-Bias—Saliency comparison GLMM (step 4) we report a parsimonious model. For model comparisons, we report LRTs which are complemented by AIC and BIC as appropriate.

## Results

The analyses are presented in four main sections. First, we report one-predictor GLMMs that assess the effects of image salience and central bias in isolation. Second, we test Central-Bias—Saliency GLMMs to explicitly address the relationship between image salience and center bias for a given saliency model. Based on these two-predictor GLMMs, we then demonstrate how individual differences and item effects can be accounted for by including random effects in the GLMMs. Next, we build a comparison GLMM, which allows for testing whether the performance differences between the three saliency models are statistically significant. For these example analyses, we specify binomial GLMMs that model whether a given image patch was fixated or not.

The fixation on the fixation cross typically extended into the period of scene presentation and fell on one of the centrally located image patches. This initial fixation was excluded from analysis. The patch it fell on was also excluded from analysis for this image and observer, irrespective of whether it was revisited or not (Nuthmann and Einhäuser, 2015)^3^.

### One-predictor models

We start by building one-predictor models which each included one of the three different saliency predictors or one of the seven different central-bias predictors as fixed effects along with the intercept. Thus, we assess how well image salience or central bias alone can be used to predict where human observers fixate. For example, the AWS-only GLMM includes AWS as the only fixed effect. In all GLMMs, the intercept represents the overall fixation probability. For saliency and/or central bias, which are both continuous input variables, the GLMM fits a linear function to the data and reports the fixed-effect regression coefficient for the slope. The slope for saliency is interpreted as the change in fixation probability (i.e., the outcome variable) associated with a one standard deviation increase in the saliency predictor. For each GLMM, we also determined the variance explained by the fixed effect(s) by calculating the marginal *R*^2^ (Nakagawa and Schielzeth, 2013; Johnson, 2014). To this end, we used the *r.squaredGLMM* function in the *MuMIn* R package (Barton, 2015).

Each GLMM included the maximal random-effects structure justified by the design (Barr et al., 2013). For the subject factor, there were two variance components (intercept and slope) and one correlation parameter for the possible correlation between random intercept and slope; the same three variance components and correlation parameters were included for the random factor “scene item.”

**Figure 3:**
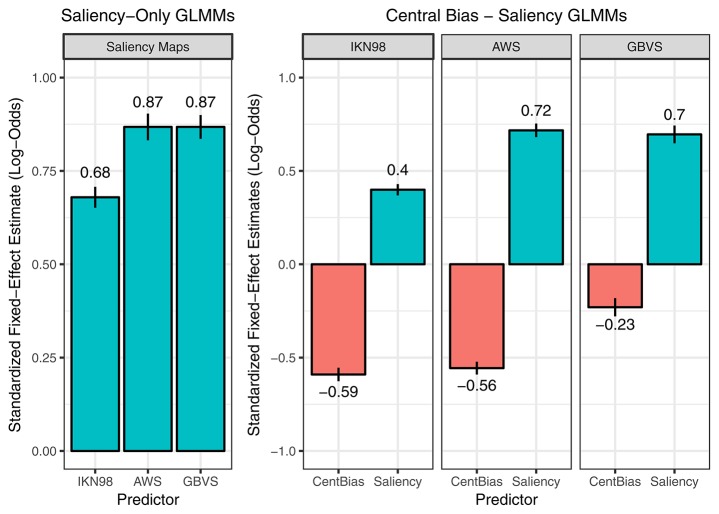
Saliency-only GLMMs (left) and Central-Bias—Saliency GLMMs (right). The figure visualizes the fixed-effect estimates and standard errors for standardized regression coefficients (i.e., slopes). The data in the left panel are from three saliency-only GLMMs that each tested a different saliency model (IKN98, AWS, GBVS). Here, a larger fixed-effects estimate for the slope corresponds to a greater effect of image saliency on fixation probability. The data on the right are from three Central-Bias—Saliency GLMMs, one for each saliency model. These GLMMs test whether image saliency has an independent effect above and beyond what can be accounted for by central bias. Each GLMM included the anisotropic Euclidean central-bias predictor, along with the intercept and the saliency predictor. Each panel in the facet plot presents the data for one saliency model. Specifically, each bar graph depicts fixed-effect estimates for standardized regression slopes for central bias (left bar) and saliency (right bar). A more negative central-bias coefficient is indicative of a stronger central bias.

The three saliency-only GLMMs each tested a different saliency model (IKN98, AWS, GBVS). For these models, Table 2 provides the parameter estimates for the fixed effects and the variance-covariance matrix of the random effects. Here, we focus on the fixed-effect estimates for the standardized regression slopes (Figure 3, left panel). For each saliency model, the corresponding GLMM showed a significant effect of local image saliency on fixation probability. As visual salience in an image patch increases, fixation probability increases as well. We can (informally) compare the strength of the saliency effect across models through the size of the standardized regression coefficient in the GLMM (Schielzeth and Forstmeier, 2009, for discussion in the context of LMMs; Schielzeth, 2010). This comparison suggests that AWS (*b* = 0.868, *SE* = 0.036, *z* = 24.27, *p* < 0.001) and GBVS (*b* = 0.868, *SE* = 0.032, *z* = 27.25, *p* < 0.001) perform equally well, with IKN98 performing the worst of the three saliency models (*b* = 0.679, *SE* = 0.028, *z* = 24.25, *p* < 0.001). Similarly, the marginal *R*^2^ was comparable for AWS (17.42%) and GBVS (17.70%) but considerably reduced for IKN98 (11.78%).

**Table 2:** Three saliency-only GLMMs fitting fixation probability for a scene memorization task.

	**B**	**SE**	**z**	**p**
**IKN98**				
**Fixed effects**				
Intercept	−1.1016	0.0292	−37.70	<0.001
Saliency	0.6795	0.0280	24.25	<0.001
**Random effects**				
Groups	Name	Variance	SD	Correlation
Subject	Intercept	0.01867	0.1366	Intercept
	Saliency	0.00321	0.0566	−0.64
Item	Intercept	0.05790	0.2406	Intercept
	Saliency	0.10249	0.3201	−0.04
**AWS**				
**Fixed effects**				
Intercept	–1.1149	0.0331	−33.71	<0.001
Saliency	0.8678	0.0358	24.27	<0.001
**Random effects**				
Groups	Name	Variance	SD	Correlation
Subject	Intercept	0.01839	0.1356	Intercept
	Saliency	0.00190	0.0436	−0.01
Item	Intercept	0.09464	0.3076	Intercept
	Saliency	0.18093	0.4254	0.14
**GBVS**				
**Fixed effects**				
Intercept	−1.1605	0.0351	−33.10	<0.001
Saliency	0.8678	0.0318	27.25	<0.001
**Random effects**				
Groups	Name	Variance	SD	Correlation
Subject	Intercept	0.02706	0.1645	Intercept
	Saliency	0.01601	0.1265	−0.80
Item	Intercept	0.08420	0.2902	Intercept
	Saliency	0.09119	0.3020	0.04

*Estimates of coefficients, standard errors, and z-ratios for fixed effects and variances, standard deviations, and correlations for random effects*.

**Figure 4:**
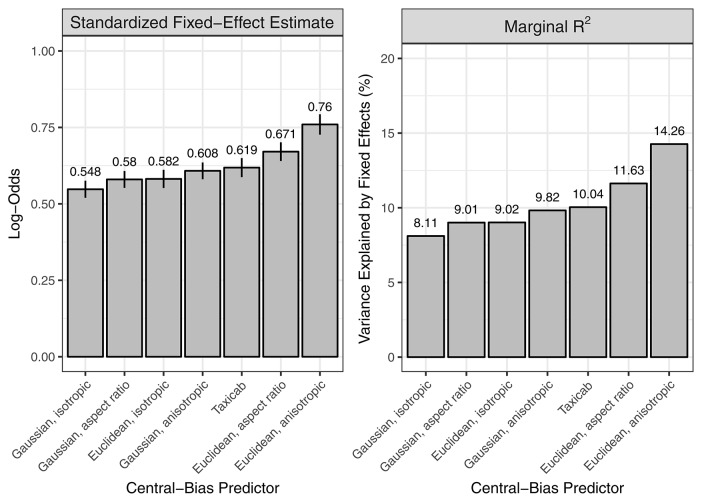
One-predictor GLMMs, each testing a dedicated central-bias variable. Two measures were used to rank seven central-bias variables: the standardized regression coefficient for the fixed effect central bias **(left)** and the marginal *R*^2^ **(right)**. For visualization purposes, the negative regression coefficients from the GLMMs are presented as absolute values.

In addition to the saliency-only GLMMs, we specified one-predictor GLMMs that each tested one of the seven central-bias predictors. We also determined the variance explained by the fixed effect central bias in a given GLMM by calculating the marginal *R*^2^. As expected, in all GLMMs the central-bias predictor showed a significant negative effect of distance to center on fixation probability; as the distance from image center increases, fixation probability decreases. We then used two measures to rank the central-bias variables: the standardized regression coefficient for the fixed effect central bias and the marginal *R*^2^. Both measures provided the same ranking, which is illustrated in Figure 4. The effect of central bias was largest in size for the anisotropic Euclidean predictor, and this predictor also explained the most variance in the outcome. The lowest values were obtained for the isotropic Gaussian predictor. As a general pattern, Euclidean variables did better than Gaussian variables, and anisotropic variables did better than aspect-ratio variables, which again did better than the isotropic variables.

### Central-bias—saliency GLMMs

**Figure 5:**
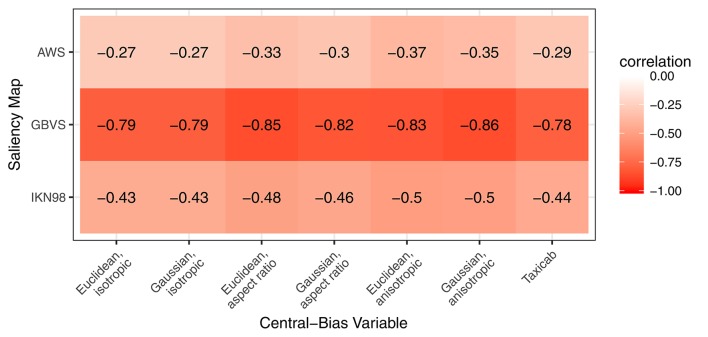
Pairwise correlation between saliency and distance from center for three different saliency maps (rows) and seven different central-bias variables (columns). Correlations are strongest for GBVS.

In the next step, we explicitly address the relationship between image salience and center bias. Averaged across images, the scenes used in our study have a modest central feature bias. In our analysis framework, the central feature bias shows in a significant negative correlation between the cells' local saliency values and their distance from scene center. For our images, Figure 5 shows the pairwise Spearman's rank correlation between saliency and distance from center for the three saliency maps (rows) and seven central-bias variables (columns). The strength of these correlations is lowest for AWS (*r* = −0.27 to *r* = −0.37, *p* < 0.001), followed by moderate correlations for IKN98 (*r* = −0.43 to *r* = −0.50, *p* < 0.001). The correlations are particularly large for GBVS (*r* = −0.78 to *r* = −0.86, *p* < 0.001), presumably because this model implicitly incorporates a center bias. Now, due to this correlation, a saliency-only GLMM can potentially yield a significant effect of salience on fixation probability even if salience was irrelevant for fixation selection. Therefore, we now specify three Central-Bias—Saliency GLMMs to test whether image salience has an independent effect above and beyond what can be accounted for by central bias. Each GLMM included the anisotropic Euclidean central-bias predictor and the relevant saliency predictor as fixed effects, along with the intercept. The maximal variance-covariance matrix of the random effects comprised 12 variance components and correlation parameters. For the item factor, there were three variance components (intercept, central-bias effect, saliency effect) and three correlation parameters for the possible correlations between each pair of these three components. For the subject factor, the same six variance components and correlation parameters were included^4^.

The results are summarized in Table 3 and Figure 3 (right panel). For all three saliency models, both central bias and saliency can independently predict whether image patches are fixated or not. The effect of saliency was again similar in size for AWS and GBVS (AWS: *b* = 0.717, *SE* = 0.037, *z* = 19.64, *p* < 0.001; GBVS: *b* = 0.696, *SE* = 0.047, *z* = 14.66, *p* < 0.001). The effect of saliency was again considerably smaller for IKN98 (*b* = 0.399, *SE* = 0.030, *z* = 13.33, *p* < 0.001). On the other hand, the effect of central bias was comparable in size for IKN98 and AWS (IKN98: *b* = −0.590, *SE* = 0.036, *z* = −16.27, *p* < 0.001; AWS: *b* = −0.556, *SE* = 0.034, *z* = −16.15, *p* < 0.001). By comparison, the independent effect of central bias was much reduced for GBVS (*b* = −0.230, *SE* = 0.049, *z* = −4.71, *p* < 0.001). One way to interpret the GLMM results is that the GBVS model owes part of its prediction performance to an implicitly incorporated central bias (cf. Harel et al., 2007). As a result, the saliency predictor explains variance that would otherwise be explained by the central-bias predictor.

**Table 3:** Three Central-Bias—Saliency GLMMs fitting fixation probability for a scene memorization task.

	**B**	**SE**	**z**	**p**
**IKN98**				
**Fixed effects**				
Intercept	−1.2084	0.0335	−36.08	<0.001
Central bias	−0.5903	0.0363	−16.27	<0.001
Saliency	0.3992	0.0300	13.33	<0.001
**Random effects**				
Groups	Name	Variance	Correlation	
Subject	Intercept	0.02925	Intercept	
	Central bias	0.02194	0.82	Central bias
	Saliency	–	–	–
Item	Intercept	0.05951	Intercept	
	Central bias	0.11434	0.57	Central bias
	Saliency	0.12918	0.00	0.49
**AWS**				
**Fixed effects**				
Intercept	−1.2248	0.0382	−32.04	<0.001
Central bias	−0.5563	0.0345	−16.15	<0.001
Saliency	0.7173	0.0365	19.64	<0.001
**Random effects**				
Groups	Name	Variance	Correlation	
Subject	Intercept	0.03123	Intercept	
	Central bias	0.02602	0.83	Central bias
	Saliency	0.00250	0.43	0.39
Item	Intercept	0.10334	Intercept	
	Central bias	0.08081	0.50	Central bias
	Saliency	0.18581	0.20	0.35
**GBVS**				
**Fixed effects**				
Intercept	−1.2027	0.0366	−32.84	<0.001
Central bias	−0.2301	0.0489	−4.71	<0.001
Saliency	0.6956	0.0474	14.66	<0.001
**Random effects**				
Groups	Name	Variance	Correlation	
Subject	Intercept	0.03102	Intercept	
	Central bias	0.01327	0.73	Central bias
	Saliency	0.00585	−0.63	−0.36
Item	Intercept	0.08580	Intercept	
	Central bias	0.30123	0.35	Central bias
	Saliency	0.30598	0.25	0.82

*Estimates of coefficients, standard errors, and z-ratios for fixed effects and variances and correlations for random effects*.

### Random effects: more than nuisance parameters

The main purpose of the GLMMs is to estimate fixed effects and to test their significance. In this framework, we have modeled subjects' and items' deviations from the fixed effects to protect against Type I errors (Schielzeth and Forstmeier, 2009; Barr et al., 2013). At the same time, including by-subject and by-item random effects is also a way of accounting for individual differences and item effects.

We demonstrate this by using the Central-Bias—Saliency GLMM for Adaptive Whitening Saliency from the previous section as an example. To recapitulate, parameters of the GLMM comprise the fixed-effects estimates and the variances and covariances of random effects. The random effects are the differences between individual coefficients (for subjects and items) and fixed effects, and have a mean of zero. Mixed models join the measurements of each subject or item together in determining how much subjects or items differ from each other (Gelman and Hill, 2007). To ascertain the reliability of individual differences and item effects, the maximal GLMM can be tested against reduced models that are nested within the maximal GLMM in that they differ in the random effects part.

#### Individual differences

**Figure 6:**
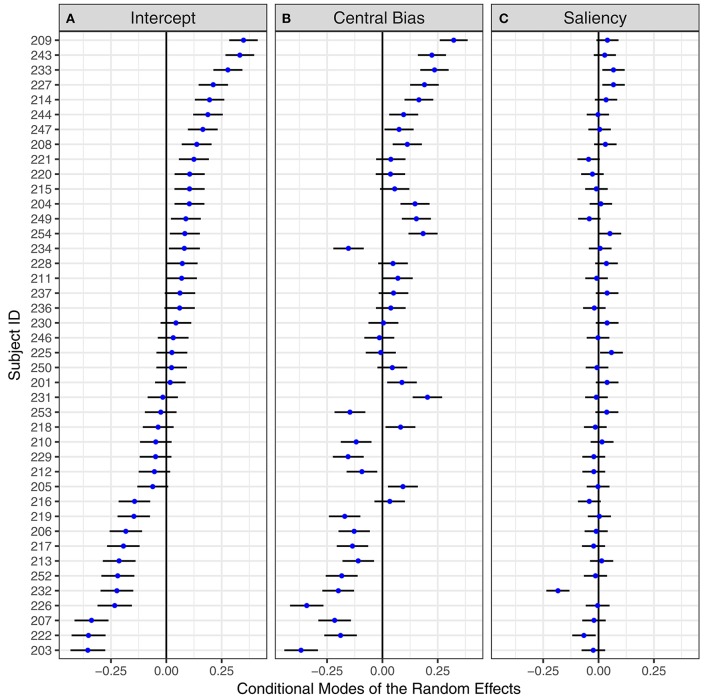
By-subject random effects for the Central-Bias—Saliency GLMM testing Adaptive Whitening Saliency. “Caterpillar plots” for conditional modes and 95% prediction intervals of 42 subjects for **(A)** mean fixation probability, **(B)** central-bias effect, and **(C)** saliency effect. Subjects are ordered by **(A)**. The numbers on the y-axis are the subject IDs.

Based on the model estimates, “predictions” (conditional modes) for subject-specific intercepts, the central-bias effect, and the saliency effect can be computed. Figure 6 displays these conditional modes for the 42 subjects, sorted by the effect for the intercept. The horizontal error bars depict 95% prediction intervals based on the evaluation of the conditional modes and the conditional variances of the random effects given the observed data (Bates, 2010; Kliegl et al., 2011). There are three noteworthy results. First, individual differences exist for the overall fixation probability (intercept) and also for the central-bias effect. There are subjects whose prediction intervals are completely on opposite sides of the zero line, which represents the corresponding fixed-effect estimate. Second, the first two panels in Figure 6 reveal a fairly consistent ordering of subjects' central-bias effects relative to their intercept effects. This positive correlation, estimated as 0.83 in the GLMM (Table 3), substantiates that the more patches were fixated, the smaller the central fixation bias (Nuthmann and Einhäuser, 2015)^5^. Third, prediction intervals for subjects' saliency effects overlap very strongly and oftentimes include the zero line. This suggests that subjects do not vary that much in their response to image salience. Nevertheless, a GLMM without variance/covariance components for the saliency effect fits significantly worse than the complete model [logLik Δ$\chi_{\left(3\right)}^{2}$ = 50.21, *p* < 0.001].

#### Item effects

**Figure 7:**
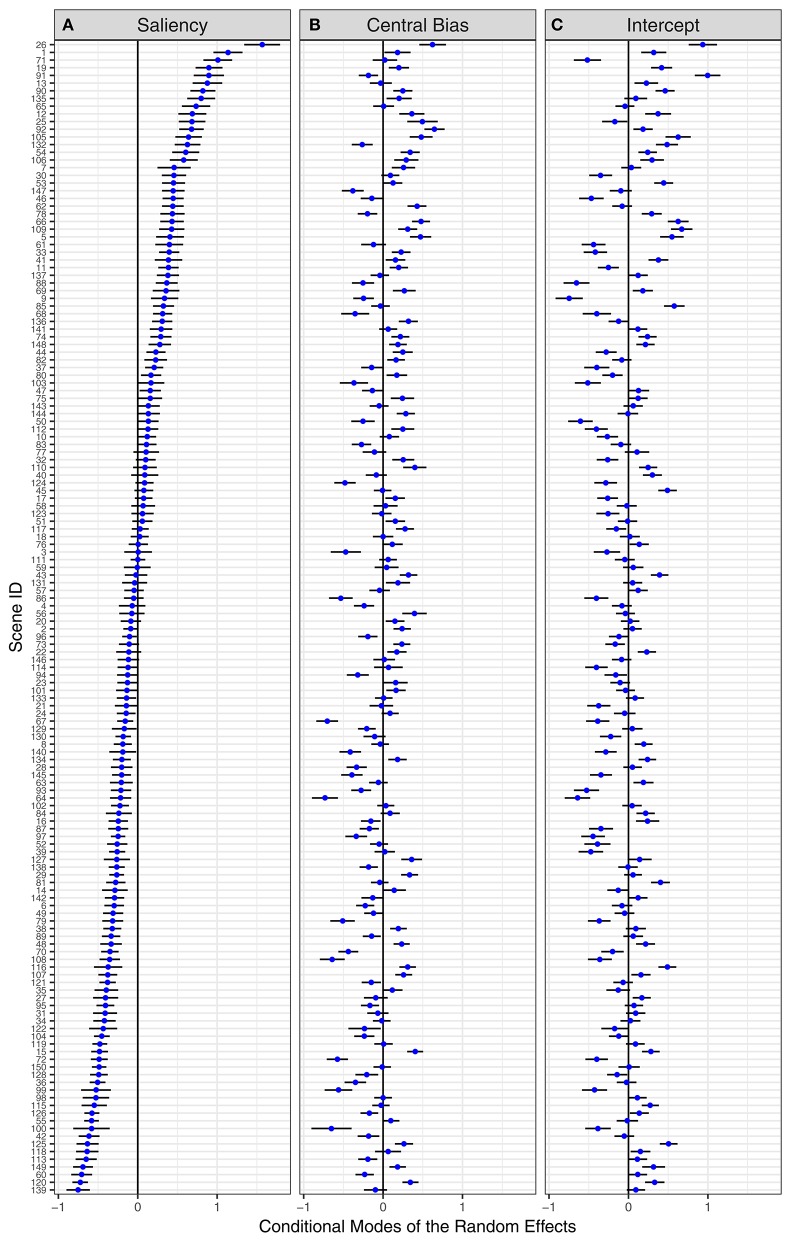
By-item random effects for the Central-Bias—Saliency GLMM testing Adaptive Whitening Saliency. “Caterpillar plots” for conditional modes and 95% prediction intervals of 150 scene items for **(A)** saliency effect, **(B)** central-bias effect, and **(C)** mean fixation probability. Scene items are ordered by the saliency effect. The numbers on the y-axis are the scene IDs.

Given the great variation in the composition of natural scenes, inferences about the role of image salience are likely to depend on the choice of scenes. Previously, it has been shown that effects of saliency (Borji et al., 2013a) and central bias (Wilming et al., 2011) on fixation selection depend on image category. Within the present approach, differences between scene categories may be accounted for by including scene category as a fixed effect in the GLMM. In the present experiment, we did not aim for testing scenes from different categories. Therefore, we chose to account for between-item differences by including by-item random effects and correlation parameters (as part of the variance-covariance matrix). Figure 7 displays the resulting conditional modes for the 150 scene items, sorted by the saliency effect. We observe considerable by-item differences on all three fixed effects (from left to right: saliency, central bias, intercept). For the saliency effect, large positive values are indicative of a particularly strong effect of image salience on fixation selection. In contrast, large negative values represent scenes for which the saliency model performs particularly poorly.

**Figure 8:**
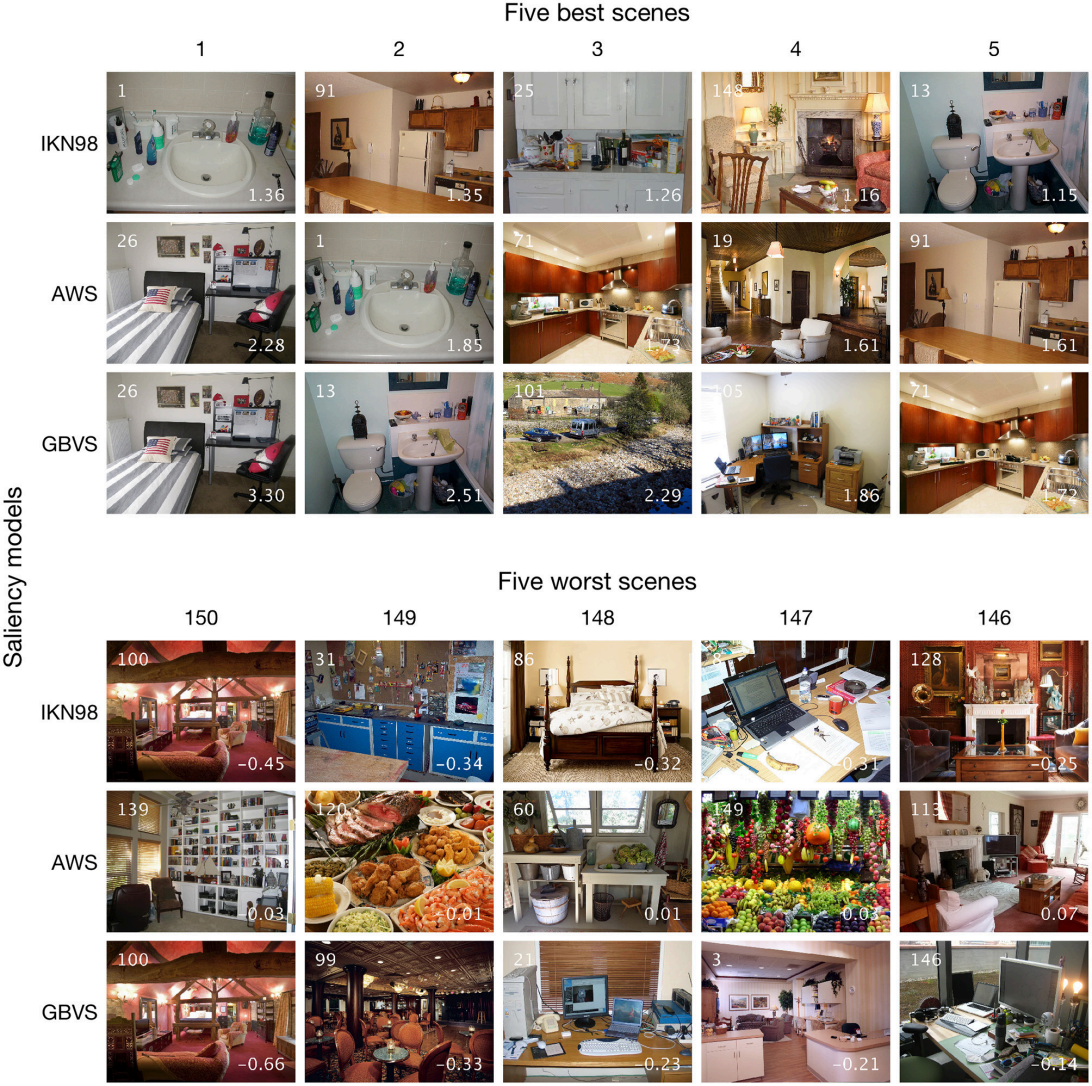
Five best and five worst scenes based on by-item random slopes for image salience. The ranking (1–5 and 150–146) is based on the results from the three Central-Bias—Saliency GLMMs (Table 3). Rows contrast the three tested saliency models. The three top rows show the five best scenes for a given saliency model; the five worst scenes are depicted in the bottom half of the figure. For a given thumbnail image, the white inset number at the top left is the scene ID. The numbers at the bottom right are the individual item coefficients, which are obtained as the sum of fixed-effect estimates and predictions for random effects. Note that there are common scenes for which the tested saliency models perform particularly well.

To illustrate the by-item saliency effects, Figure 8 shows the five best and five worst scenes according to the Central-Bias—Saliency GLMMs. The numbers at the bottom right of each thumbnail image are the individual item coefficients, which are obtained as the sum of fixed-effect estimates and predictions for random effects (Gelman and Hill, 2007). According to Figure 7, for AWS the best scene is #26 (top of Figure 7A) and the worst scene is #139 (bottom of Figure 7A). Interestingly, for scene #139 the coefficient for the saliency effect is effectively zero (coefficient = fixed effect + random effect; −0.03 = 0.72 ± 0.75), which means that salience has no impact on fixation selection for this scene. In addition to presenting results for AWS, Figure 8 also depicts the five best and five worst scenes for IKN98 and GBVS. The three saliency models are contrasted in different rows of the figure. Clearly, there are common scenes for which the tested saliency models perform particularly well (e.g., #1). However, even though the IKN98 model performs particularly well on scene #1, the AWS model still performs better on this scene, as is evident from the larger individual item coefficient. This is because AWS performs better than IKN98 overall, as expressed in the larger estimate for the fixed effect saliency (Table 3).

One reason for the relative success of saliency models in predicting human gaze behavior is in their ability to predict the location of objects, which in turn attract attention and the eyes (Einhäuser et al., 2008; Elazary and Itti, 2008; Nuthmann and Henderson, 2010; Stoll et al., 2015). This is particularly true for models like AWS, which generates some notion of objecthood using proto-objects and whitening (Garcia-Diaz, 2011).

For the scenes on which the saliency models performed very poorly, we visually inspected the images, saliency maps, and fixation data. We could make out three factors that contributed to poor model performance. First, models perform poorly if the scene contains many items of similar low-level salience, especially when their individuation is hard (e.g., scene #149, which contains many colored fruit). Second, models fail to predict fixations on objects that distinguish themselves from the background by features the model has no access to (e.g., the gramophone in scene #128 has nearly the same physical color as its surround, but its apparent shininess makes it stand out for human observers). Third, structures that differ from their surround but mainly frame a part of the scene are not fixated as often as predicted by the models (e.g., the framework in scene #100, which most models judge to be highly salient, but human fixations are instead attracted to the centers of the framed regions). As a result, some scenes (e.g., scene #100) even have negative individual item coefficients, which implies that high saliency was associated with fewer fixations.

#### Reliability of individual differences and item effects

The reliability of between-subject and between-item effects can be assessed through model comparison using LRTs. The number of possible random effects structures increases with the complexity of the design. Rather than testing all possibilities, we contrast our maximal model with two models that have gained popularity in the literature: the zcp model and the random intercept model (cf. Bates et al., 2015a). For demonstration purposes, we will again use the Central-Bias—Saliency GLMM for Adaptive Whitening Saliency. Compared with our maximal model, the zcp model estimates the same six variance components, but it does not estimate the six correlation parameters.

According to the LRT, the maximal model provided a significantly better goodness of fit than the zcp model [logLik Δ$\chi_{\left(6\right)}^{2}$ = 76.55, *p* < 0.001], suggesting that there are appreciable (non-zero) correlations between random effects. In addition, both AIC and BIC were smaller for the maximal model than for the zcp model (AIC: 286242–286335 = −93; BIC: 286400–286431 = −31). On the other hand, the zcp model provided a significantly better goodness of fit than the random intercept model [logLik Δ$\chi_{\left(4\right)}^{2}$ = 7,649.2, *p* < 0.001]; in addition, both AIC and BIC were much smaller for the more complex zcp model (AIC: 286335–293976 = −7641; BIC: 286431–294029 = −7598). Thus, the random intercept model is not adequate for our data.

### Central-bias—saliency comparison GLMM

The results from the central-bias—saliency GLMMs suggest the following rank order of saliency models: IKN98 performs least well; AWS and GBVS perform better with little difference between the two. While these analyses are informative, they do not provide us with a direct indication of whether the performance differences between the three saliency models are statistically significant. To achieve this direct comparison, we specify a GLMM which additionally includes “saliency map” as a categorical predictor. For this factor, the GLMM compares each level of the variable to a reference group; that is, one of the saliency models. We chose AWS as the reference, which allowed for testing two important group differences. First, fixation probability for IKN98 can be compared to AWS. Second, we can test for differences between AWS and GBVS. As before, the GLMM included image saliency and central bias as fixed effects. The new addition is that group differences are tested through interactions.

Most importantly, by including the interaction of local saliency (continuous predictor) and saliency map (categorical predictor), the GLMM will first test the effect of local saliency on fixation probability for the reference saliency map (AWS), which will be reported as a simple effect. In addition, the GLMM will test whether this effect was significantly different for either of the other two saliency maps (interactions). The actual coefficient for the effect of saliency in the IKN98 saliency model (or GBVS) can be derived by summing the simple effect coefficient and the relevant interaction coefficient. Similarly, the fixed effect for central bias will test the independent effect of central bias for the reference saliency map (AWS). The interaction between central bias and saliency map will test whether the effect of central bias was significantly different for either of the other two saliency maps. Taken together, the comparison GLMM includes seven fixed effects (intercept, two main effects, four interaction coefficients).

**Figure 9:**
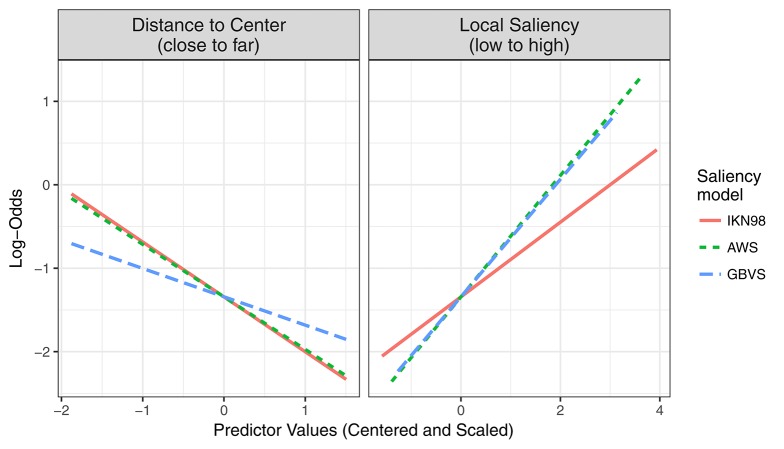
Central-Bias—Saliency Comparison GLMM. The figure visualizes predicted partial effects of central bias **(left)** and image saliency **(right)** on fixation probability in log-odds scale for three saliency models that were included in the comparison (IKN98, red solid line; AWS, green dashed line; GBVS, blue long-dashed line).

For this GLMM, the maximal random-effects structure would require estimating 56 parameters (by item: random intercept, 6 random slopes, 21 correlation terms; by subject: same as by item). Since the maximal model did not converge, we set the correlation parameters to zero (Barr et al., 2013; Bates et al., 2015b). The full random-effects structure of the zcp model required 14 variance components to be estimated. For this model, the variances for four by-subject random effects were estimated as zero. These were the difference scores describing how subjects' responses to IKN98 or GBVS differed from their responses to AWS. Those four random effects were excluded from the final model. The results for the final model are summarized in Table 4. Moreover, Figure 9 depicts the predicted partial effects of central bias (left panel) and saliency (right panel) on fixation probability for the three tested saliency models. The partial effect describes the effect of the independent variable in question when all other variables in the model are statistically controlled. For example, for computation of the partial saliency effect the fixed effect capturing the central bias was removed, as was any between-subject and between-item variance. GLMM predictions were extracted using the *keepef* function from the *remef* package (version 1.0.6.9, Hohenstein and Kliegl, 2014).

**Table 4:** Estimates of coefficients, standard errors, and z-ratios for fixed effects and variances for random effects for the comparison GLMM in which the effects of saliency and central bias were simultaneously evaluated for three different saliency maps.

**Fixed effects**				**Random effects, Variance**	
**Predictor**	**B**	**SE**	***z***	**By-items**	**By-subjects**
Intercept	−1.2157	0.0333	−36.46	0.05601	0.03091
Central bias (AWS)	-0.5700	0.0329	−17.33	0.07742	0.02284
Central bias: IKN98-AWS	−**0.0264**	**0.0162**	−**1.63**	0.03122	-
Central bias: GBVS-AWS	0.2932	0.0310	9.44	0.13229	-
Saliency (AWS)	0.6622	0.0329	20.10	0.15397	0.00124
Saliency: IKN98-AWS	−0.2688	0.0262	−10.24	0.09445	-
Saliency: GBVS-AWS	−**0.0279**	**0.0340**	−**0.82**	0.16012	-

*Number of observations: 887,172; groups: scene items: 150, subjects: 42. Non-significant coefficients are set in bold (|z| < 1.96, p > 0.05)*.

Regarding the central-bias effect, there was no significant difference between AWS and IKN98 (*b* = −0.026, *SE* = 0.016, *z* = −1.63, *p* = 0.10). However, the central-bias slope was significantly less negative for GBVS than for AWS (*b* = 0.293, *SE* = 0.031, *z* = 9.44, *p* < 0.001). The actual coefficient for the independent effect of central bias for GBVS was −0.277 (−0.570 + 0.293). Relative to AWS (*b* = 0.662, *SE* = 0.033, *z* = 20.10, *p* < 0.001), saliency had a significantly reduced effect on fixation probability for IKN98 (*b* = −0.269, *SE* = 0.026, *z* = −10.24, *p* < 0.001). For GBVS, the saliency effect was not significantly different from AWS (*b* = −0.028, *SE* = 0.034, *z* = −0.82, *p* = 0.41).

### Control analyses

For the three central-bias—saliency GLMMs, each testing one of the saliency maps, we performed control analyses to explore (a) the different central-bias predictors, (b) different grid cell sizes, and (c) different random-effects structures. Regarding the resolution of the grid, we repeated the analyses for both a fine 16 × 12 grid as well as a coarse 4 × 3 grid (cf. Nuthmann and Einhäuser, 2015). For all three grid resolutions, including the default 8 × 6 grid, we repeated the analysis for each of the seven central-bias predictors. For each of these GLMMs, we tested three different random-effects structures: (a) maximal model, (b) zcp model, and (c) the random intercept model. Altogether, 189 central-bias—saliency GLMMs were specified.

A systematic comparison is beyond the scope of this article. Instead, we report an informal summary of our observations. IKN98 consistently performed the worst of the three tested saliency models. For the GBVS model with its implicitly built-in central bias, performance depended on the chosen central-bias predictor in the following way: for six out of seven central-bias predictors, the independent effect of the central-bias predictor was either completely gone or even changed sign; in both cases, GBVS outperformed AWS. By comparison, for the best-performing (Figure 4) anisotropic Euclidean central-bias predictor the independent (negative) effect of central bias was strongly diminished and AWS and GBVS kept performing equally well (Figure 3, right panel).

When the random-effects structure was varied, the zcp model and the maximal model consistently yielded very similar results. Including random slopes along with the random intercepts consistently increased the standard errors of fixed-effects estimates. To be clear, the effects of central bias and saliency were significant in all GLMMs. However, in the random intercept models the fixed-effects estimates were attenuated with less standard error (cf. Demidenko, 2013), due to the models not including random slopes for critical effects. Thus, for the present eye-movement dataset random intercept models are likely to be anticonservative when comparing saliency models for which performance differences are minimal.

## Discussion

Given the large number of computational models of visual salience that exist in the literature, a key question is: how can one fairly evaluate all these models? In this article, we propose a new method which has desirable properties, discussed below. Our approach makes use of generalized linear mixed modeling, a powerful statistical technique which has become increasingly common in psychology and psycholinguistics, but is not yet widely used in computational vision and modeling.

### Baseline and reference frame

For existing evaluation methods, the choice of an appropriate baseline is a non-trivial issue to address (Bylinskii et al., 2016a). An important advantage of our method is that there is no requirement to define a baseline (Nuthmann and Einhäuser, 2015, for discussion).

Wilming et al. (2011) suggested that—along with the performance measure—researchers should report the lower and upper bound for model performance as a reference frame. Specifically, the authors propose that the predictive power of the image- and subject-independent central bias constitutes a (challenging) lower bound for model performance, which “any useful model has to exceed” (p. 10). Moreover, they use the consistency of selected fixation locations across different subjects (inter-subject consistency) as an upper bound for model performance, following other authors (e.g., Peters et al., 2005; Harel et al., 2007; Einhäuser et al., 2008; Kanan et al., 2009). At the same time, Wilming et al. show that it is possible to surpass the upper bound by combining subject- and image-specific information.

### New approach using grid and GLMM

Here, we introduce a different (and complementary) approach. We aimed to derive a method which allows for directly describing the relationship between image salience and fixation probability, after controlling for subjects' tendency to look at the center of scene images. In this framework, central bias and saliency are evaluated as predictors in advanced regression models. The central-bias-only GLMMs can be viewed as representing the lower bound of model performance. The central-bias-only GLMMs (Figure 4) can be contrasted with saliency-only GLMMs (Figure 3, left panel; Table 2). Our analysis of three saliency models and seven different central-bias variables suggested that saliency alone could predict fixation selection better than central bias alone. The one exception was the anisotropic Euclidean central-bias measure, for which the marginal *R*^2^ was larger than for the IKN98 saliency model (14.26 vs. 11.78%). Now, the problem with these analyses is that we typically observe significant correlations between saliency and distance from center (see Figure 5 for the present data). We can address this issue by specifying Central-Bias—Saliency GLMMs, which take this correlation into account and yield partial effects for central bias and saliency.

Among the saliency models that we tested, GBVS was the only model with an intrinsic central bias. A joint consideration of our main and control analyses suggests that the ranking of the GBVS model relative to the AWS model depended on which central-bias predictor was used. In the main analyses, for which we used the best-performing anisotropic Euclidean central-bias predictor, AWS and GBVS performed equally well. For all other central-bias predictors, GBVS tended to outperform AWS. While this dependency warrants further investigation, the present analyses clearly demonstrate that our method identified GBVS as a model that already incorporates a center preference to improve performance. We make the following recommendations for future research. If possible, the researcher should determine how the central bias was incorporated in a given saliency model and then select (or define) an appropriate central-bias predictor for the GLMM. It is a strength of our approach that it allows for including different forms of central bias.

### Assessing differences between groups

The present method can be extended to assess effects of viewing task, image class, or subject group by including additional fixed effects in the GLMM. For example, to make full use of our new eye-movement corpus we can analyze whether older adults differ from young adults in terms of scene coverage, central bias and—most of all—the importance of image salience for fixation selection during scene viewing. To give another example, End and Gamer (2017) used our method to assess the independent contributions of social features and physical saliency on gaze behavior.

Regarding the comparison of saliency models, the method can be expanded to more than three saliency models. The comparisons enter the GLMM as contrasts which are chosen such that they test the comparisons that are of most interest. More complex GLMMs will lead to more complex maximal random-effects structures, which may make the selection of an appropriate random-effects structure more difficult. One way to mitigate this problem would be to first test between different families of saliency models and then within the winning model family.

### Random effects for subjects and scene items

In experimental research, it is common to treat subjects as the sole random factor in the analysis (Judd et al., 2012a). However, in research on real-world scene perception, individual characteristics of the scene items cannot be ignored. One way to assess such item variance is to fit a separate model to each scene item's data. With this approach there is a risk of overfitting the data, because data points with extreme values are weighted too heavily (Gelman and Hill, 2007). In comparison, GLMMs provide enhanced precision in prediction for items, because a given model considers the behavior of any given item in the light of what it knows about the behavior of all the other items, and shrinks extreme values accordingly (Baayen, 2008). Similarly, unreliable between-subject variance in the effects is removed through shrinkage toward the estimate of the population mean (Kliegl et al., 2011; Makowski et al., 2014). Another advantage is that GLMMs allow one to generalize to both populations of subjects and items on the basis of a single analysis (Locker et al., 2007).

In our study design, subjects and items were completely crossed with each other such that every subject viewed all 150 scene images. It can be advantageous to test saliency models on data sets comprising a large number of scenes from different categories. In this case, each subject can only provide data for a subset of items. For example, the CAT2000 dataset comprises 4,000 images of which 800 were presented to a given subject (Borji and Itti, 2015). Each image was viewed by 24 different observers. GLMMs can handle data from such designs in which subjects and items are partially crossed.

Our investigation of random effects in the GLMMs showed that item variances were much larger than subject variances (cf. Nuthmann and Einhäuser, 2015). Results for by-item saliency effects suggested that the saliency models did particularly well on certain scenes, and completely failed on others. This further supports the observation that current saliency models tend to find it difficult to predict fixation selection for some particular type of stimulus (Borji et al., 2013a). Knowing on what images their saliency model failed may help researchers to make informed decisions about how to improve their model (Kümmerer et al., 2015). Moreover, the present method could be used to identify images for which particular predictors are particularly (ir)relevant, and thus guide the selection of images for experiments that best discriminate between saliency models. Finally, the analyses substantiated that the central bias is image and subject dependent.

### Classification of evaluation measures and desirable properties

A systematic comparison of our method with existing methods for the evaluation of saliency maps is beyond the scope of the present article. Instead, we will situate our method in existing classifications of evaluation measures. Moreover, we will discuss how our method fares with respect to desirable properties for evaluation measures suggested by Wilming et al. (2011).

Measures can be categorized as location-based or distribution-based depending on whether the ground truth (here: human eye fixations) is represented as discrete fixation locations or a continuous fixation map (Riche et al., 2013; Bylinskii et al., 2016a). Riche et al. (2013) further distinguish “specific metrics” and “common metrics” from “hybrid metrics.” Specific metrics are the ones that were specifically developed for the evaluation of saliency models. Here, we combine *a-priori* parcellation of scenes with a common statistical analysis method (GLMM). To address our research question, we chose to code observers' eye fixations in a grid, which allows for homogeneous, exhaustive image coverage. Thus, our method is best described as a hybrid location-based method.

Wilming et al. (2011) derived a set of four desirable properties for evaluation measures: “few parameters,” “intuitive scale,” “low data demand,” and “robustness.” First, the outcome of an evaluation measure should not depend on arbitrary parameters (Wilming et al., 2011). Parameter estimation for GLMMs does not involve any arbitrary parameters. However, the resolution of the grid is chosen by the user. We would advise against using a very small grid cell size. Theoretically, the size of the grid cells could be reduced to the pixel level. The problem is that fixation locations are too sparse to directly apply pixel-wise statistical modeling (Lao et al., 2017). Therefore, fixation data need to be smoothed to apply pixel-wise modeling. However, this approach similarly introduces an arbitrary scale, that is the width of the smoothing kernel (e.g., the standard deviation of a Gaussian filter), which is no more justified on theoretical grounds than choosing a cell size for scene parcellation. In practice, the same heuristics apply to choosing filter kernels and grid sizes: it is not advisable to choose a size substantially smaller than the measurement precision, and the size should not exceed the typical scale of the scene properties or features of interest.

The second desirable property for a good evaluation measure is an intuitive scale. Specifically, the measure should inform about the quality of the prediction, including deviation from optimal performance (Wilming et al., 2011). GLMMs are regression techniques. To make the continuous predictors central bias and saliency comparable within and across models, we standardized their units to units of standard deviations. Now that predictors are placed on a common scale (i.e., they are commensurate), we can compare the strength of effects within and across models through the size of the standardized regression coefficients (Figures 3, 4, 9). Moreover, the marginal and conditional *R*^2^ for GLMM (Nakagawa and Schielzeth, 2013; Johnson, 2014) provide information about the absolute model fit in terms of variance explained by the model. Marginal *R*^2^ determines variance explained by fixed effects, and conditional *R*^2^ gauges variance explained by both fixed and random effects. *R*^2^ ranges from 0 to 1 where 1 represents a perfect fit. In addition, information criteria like the AIC and BIC provide an estimate of the relative fit of alternative models.

The third criterion “low data demand” addresses the fact that observers make a limited number of saccades when exploring images of naturalistic scenes (Wilming et al., 2011). In our method, we use fixated grid cells as positive instances. In principle, reliable estimates for the GLMM parameters can be computed on relatively few data points. Of course, the proportion of fixated grid cells depends on how explorative an individual subject's viewing behavior is. In our analyses, less explorative individuals had a reduced intercept and increased central bias (Table 3, Figure 6). In addition, the degree of exploration was found to vary across scene items (Table 3, Figure 7). The proportion of fixated grid cells is likely to depend on the trial duration as well. The number of saccades to different scene regions tends to increase with increasing trial duration. In the present experiment, scenes were presented for 6 s. For datasets from static scene viewing that are included in the MIT Saliency Benchmark (http://saliency.mit.edu), presentation duration ranges between 3 s (Borji and Itti, 2015) and 15 s (Le Meur et al., 2006). Finally, we remind the reader that the resolution of the grid—set by the user—will affect the sparsity (fine grid) or density (coarse grid) of the observation matrix.

The fourth criterion is “robustness,” which means that a measure should not be dominated by single extreme values (Wilming et al., 2011). GLMMs provide robust measurements in the sense that unreliable between-subject and between-item variance is removed through shrinkage toward the estimate of the population mean (Kliegl et al., 2011; Makowski et al., 2014). In summary, our method scores well with respect to properties that are desirable for evaluation methods.

### Future directions

To describe our method, we used three saliency models. Future studies should aim to test a larger set of saliency models on different datasets. Moreover, it will be informative to systematically compare our method with existing ones.

During the last 10 years, saliency models have begun to diverge into two different classes: models of *fixation prediction* and models of *salient object or region detection* (Itti and Borji, 2014). Fixation prediction models compute a saliency map that is thought to simulate observers' eye fixations over the scene, and their performance can be evaluated with the method proposed here. Salient object detection or salient region detection models, on the other hand, attempt to segment the most salient object or region in a scene (Borji, 2015, for review). Given the inherent correlation between salient locations in a salient map and objects (e.g., Nuthmann and Henderson, 2010), a systematic relationship between eye fixations and salient objects is conceivable (Li et al., 2014). Future extensions of our approach will facilitate this research in several ways. First, the GridFix toolbox can be extended to accommodate irregular regions of interest (e.g., object outlines). Second, object-based GLMMs can then be used to test the hypothesis that highly salient objects are indeed the ones that attract the most fixations. In our own research, we have used this approach to test an alternative role of image salience: rather than prioritizing locations, salience aids prioritization among objects (Stoll et al., 2015).

Currently, GridFix facilitates analysis of fixation probability through calculating either a binary variable (1 fixated, 0 not fixated) or fixation counts. In a future extension, one could incorporate the *temporal* aspect of eye guidance in scenes (cf. Nuthmann, 2017, for review) by coding fixation *times* associated with the grid cells or object outlines. Analyzing any measure of fixation time equates to analyzing a continuous dependent variable, which requires using LMM rather than GLMM.

## Conclusion

Central bias has been identified as a major challenge in assessing the performance of saliency models. Addressing this issue, we introduced a new method for model evaluation and comparison which combines *a-priori* parcellation of scene images with GLMM. This approach allows for assessing how well saliency models predict where human observers fixate in naturalistic images, above and beyond the central bias. Interobserver differences and image dependencies can be captured through random effects in the GLMMs. The GridFix toolbox facilitates the application of our method.

## Author contributions

All authors designed the research. AN analyzed the data and prepared the figures. All authors interpreted the data. AN drafted the manuscript, and WE and IS provided critical revisions. IS implemented an open-source Python toolbox for data processing. All authors approved the final version of the manuscript for submission.

### Conflict of interest statement

The authors declare that the research was conducted in the absence of any commercial or financial relationships that could be construed as a potential conflict of interest.

## Footnotes

^1^The GridFix toolbox is available at https://zenodo.org/record/998554.

^2^Note that *v* = 1 for isotropic central-bias variables.

^3^Two situations are possible. The first one is that the patch was only fixated during the extended fixation on the pre-trial fixation cross. The second possibility is that the patch additionally received an immediate refixation or later revisit. Since the initial fixation is routinely excluded, the first case would be coded as “not fixated,” the second one as “fixated.” It can be debated whether coding the first case as “not fixated” is appropriate, as the patch was fixated for some period of time during the fixation on the pre-trial marker. Therefore, GridFix offers to exclude this patch from analysis as a clean solution. Specifically, by default the patch is coded as missing value (NaN) and thereby ignored in subsequent analyses.

^4^For IKN98, the by-subject random effect for saliency was excluded as its variance was close to zero, leading to convergence problems.

^5^The fixed-effects estimate for central bias is negative. A positive conditional mode of the central-bias effect translates to a less negative central-bias coefficient for this subject, which is indicative of a weaker central bias.
